# Arthroscopic Management of an Unusual Fibrofatty Loose Body Presumed to Arise From Hoffa’s Fat Pad: A Case Report

**DOI:** 10.7759/cureus.116435

**Published:** 2026-09-17

**Authors:** John A Spallanzani, Henry K Mah, Jamil Ayinla, Tarek S Abd-Allah, Matthew Wert

**Affiliations:** 1 Internal Medicine, Wyckoff Heights Medical Center, New York City, USA; 2 Obstetrics and Gynecology, Wyckoff Heights Medical Center, New York City, USA; 3 Surgery, Brookdale University Hospital and Medical Center, New York City, USA; 4 Orthopedic Surgery, NewYork–Presbyterian Brooklyn Methodist Hospital, New York City, USA

**Keywords:** arthroscopy knee, hoffa’s fat pad, infrapatellar fat pad, intra-articular loose body, plica syndrome

## Abstract

We report the case of a 31-year-old man with persistent anterior knee pain and mechanical locking and catching despite conservative management. Preoperative magnetic resonance imaging (MRI) demonstrated an intra-articular loose body in the patellofemoral compartment with associated lateral meniscal and synovial abnormalities. Arthroscopy identified a greater than 5-mm fibrofatty loose body whose gross appearance was considered suggestive of a detached remnant of Hoffa's fat pad. Histopathologic analysis was not obtained, and direct continuity with the infrapatellar fat pad was not documented; therefore, the tissue of origin remains presumed. The loose body was removed with suction and arthroscopic forceps, and associated plica, synovial, and lateral meniscal pathology was treated concurrently. At six weeks, the patient demonstrated marked short-term improvement after combined arthroscopic treatment, with a visual analog scale pain score decreasing from 7/10 to 1/10, resolution of locking, and improvement in range of motion from 10°-110° to 0°-130°. This case illustrates the diagnostic uncertainty that may accompany unusual intra-articular fibrofatty lesions and highlights the complementary roles of MRI and arthroscopy when persistent mechanical symptoms warrant operative evaluation.

## Introduction

Hoffa's fat pad, or the infrapatellar fat pad, is an intracapsular but extrasynovial structure posterior to the patellar tendon that contributes to knee joint lubrication and biomechanical function [1]. Pathology involving the infrapatellar fat pad can cause anterior knee pain and mechanical symptoms, but its clinical presentation is nonspecific and may overlap with meniscal, patellofemoral, and other intra-articular disorders [2].

Published reports involving Hoffa's fat pad more commonly describe impingement, fibrosis, chondromatous or ossified lesions, and other tumor-like processes rather than a detached nonossified fibrofatty loose body [3-5]. The incidence of a detached fibrofatty loose body presumed to originate from the infrapatellar fat pad has not been established. Such a lesion should be distinguished from more common intra-articular loose bodies or masses, including osteochondral or meniscal fragments, synovial chondromatosis, para-articular chondroma, lipomatous lesions, and tenosynovial giant-cell tumors. Proposed mechanisms involving chronic impingement, inflammation, repetitive microtrauma, or fibrosis remain theoretical rather than established for a detached fibrofatty fragment [1,6].

This case describes the MRI and arthroscopic evaluation of an unusual fibrofatty intra-articular loose body in the patellofemoral compartment whose gross appearance was considered suggestive of a presumed Hoffa's fat-pad remnant. The case emphasizes diagnostic uncertainty in the absence of histopathologic confirmation and the importance of correlating imaging findings with arthroscopic appearance and other intra-articular pathology.

## Case presentation

A 31-year-old male with a body mass index of 28 kg/m² presented with a six-month history of right anterior knee pain associated with mechanical locking and catching following an injury during a soccer game. Symptoms persisted despite a course of nonoperative management that included physical therapy, nonsteroidal anti-inflammatory medication, and activity modification. The available source documentation did not provide sufficient detail to reliably report the duration of each individual conservative modality, prior knee trauma, activity level, or additional relevant medical and surgical history; these details were, therefore, not retrospectively inferred.

Clinical examination demonstrated tenderness over the anterior knee, a positive McMurray test, and limited range of motion from 10° to 110°. The available preoperative documentation did not reliably report effusion, joint-line tenderness, patellofemoral provocative signs, ligamentous testing, or the presence or absence of a palpable mass; these findings were therefore not reconstructed retrospectively.

Preoperative magnetic resonance imaging (MRI) of the right knee was performed without intravenous contrast using coronal proton-density (PD), coronal fat-suppressed T2, sagittal proton-density, sagittal fat-suppressed T2, axial fat-suppressed PD, and sagittal T2 anterior cruciate ligament (ACL) sequences. MRI demonstrated a flattened hypointense triangular lesion measuring approximately 1.4 × 0.5 × 0.2 cm located anterior to the anterior cruciate ligament, contiguous with adjacent scarring of Hoffa's fat pad (Figures 1A, 1B). The lesion was associated with trace knee effusion and lateral meniscal abnormalities. Although MRI suggested a fat-containing lesion arising near the infrapatellar fat pad, the precise tissue origin could not be established preoperatively.

**Figure 1:**
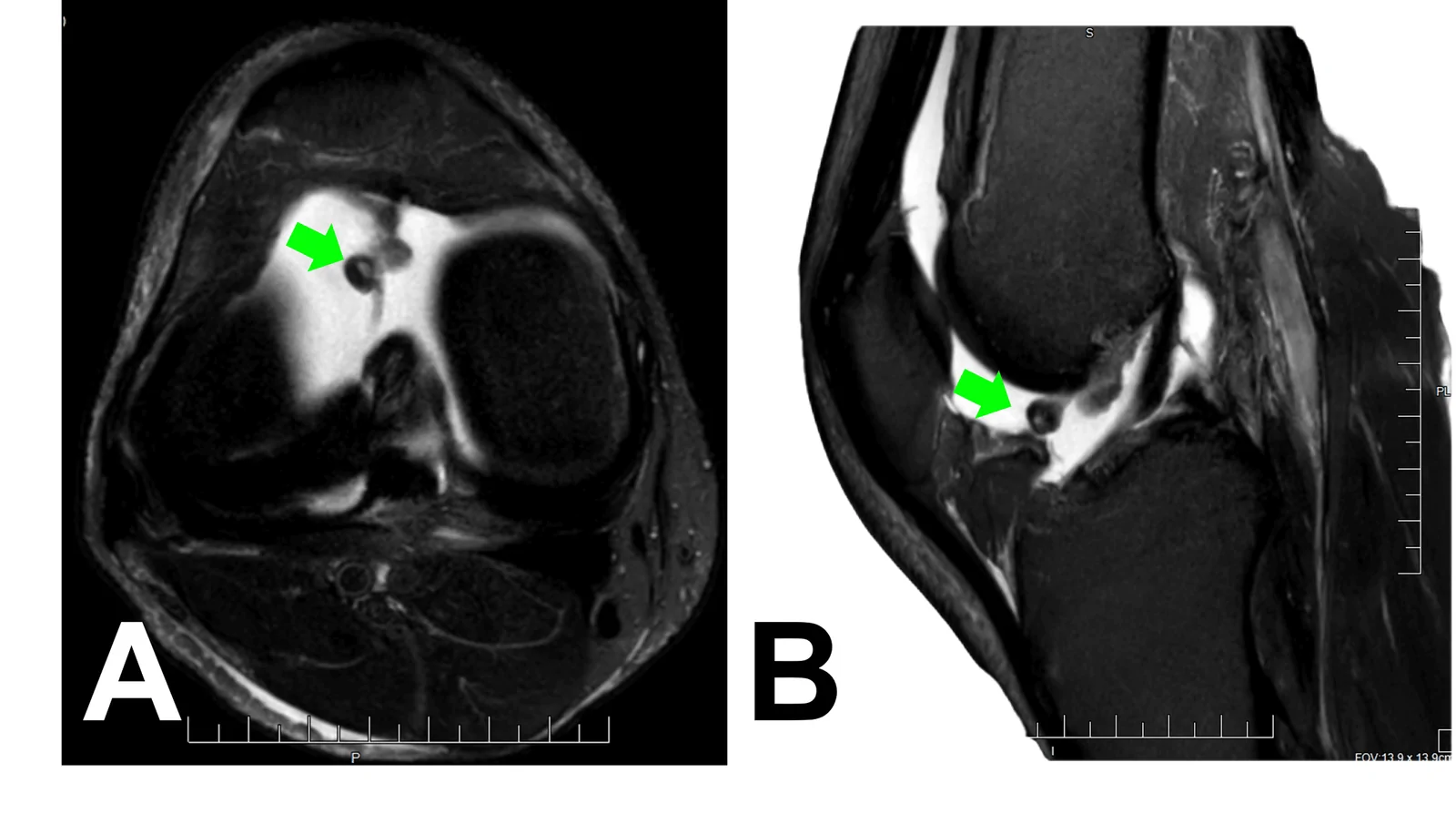
Preoperative MRI demonstrating a suspected loose body within the patellofemoral compartment of the right knee. Preoperative MRI of the right knee demonstrating a flattened hypointense triangular lesion (approximately 1.4 × 0.5 × 0.2 cm; green arrow) anterior to the anterior cruciate ligament and contiguous with adjacent Hoffa's fat-pad scarring. Images were obtained using axial (A) fat-suppressed proton-density and sagittal (B) fat-suppressed T2 sequences without intravenous contrast.

Following discussion of the risks, benefits, and alternatives, the patient provided informed consent for right knee arthroscopy. Written informed consent was also obtained for publication of this case report and the accompanying images.

After informed consent was obtained, the patient was correctly identified in the preoperative holding area, and the operative knee was marked with the surgeon's initials to confirm laterality. The patient was transported to the operating room and positioned supine. General anesthesia was induced, a formal surgical time-out was performed, and prophylactic intravenous antibiotics were administered. The right lower extremity was prepared and draped in the standard sterile fashion with the thigh secured in an arthroscopic leg holder, and a thigh tourniquet was used.

A standard anterolateral viewing portal was established using a #11 blade, and a 30° Stryker arthroscope (Stryker, Kalamazoo, MI, USA) was introduced into the knee joint. Under direct visualization, an anteromedial working portal was established using spinal-needle localization. A systematic diagnostic arthroscopy was then performed with sequential evaluation of the suprapatellar pouch, patellofemoral joint, medial compartment, lateral compartment, and intercondylar notch.

A loose body measuring greater than 5 mm was identified within the patellofemoral compartment and removed using suction and arthroscopic forceps (Figures 2A-2D). The lesion demonstrated a fibrofatty gross appearance and was described intraoperatively as a remnant of Hoffa's fat pad. Histopathologic examination was not obtained, and the operative report did not document a discrete donor site or direct continuity between the loose body and the infrapatellar fat pad; therefore, its tissue of origin remains presumed.

**Figure 2:**
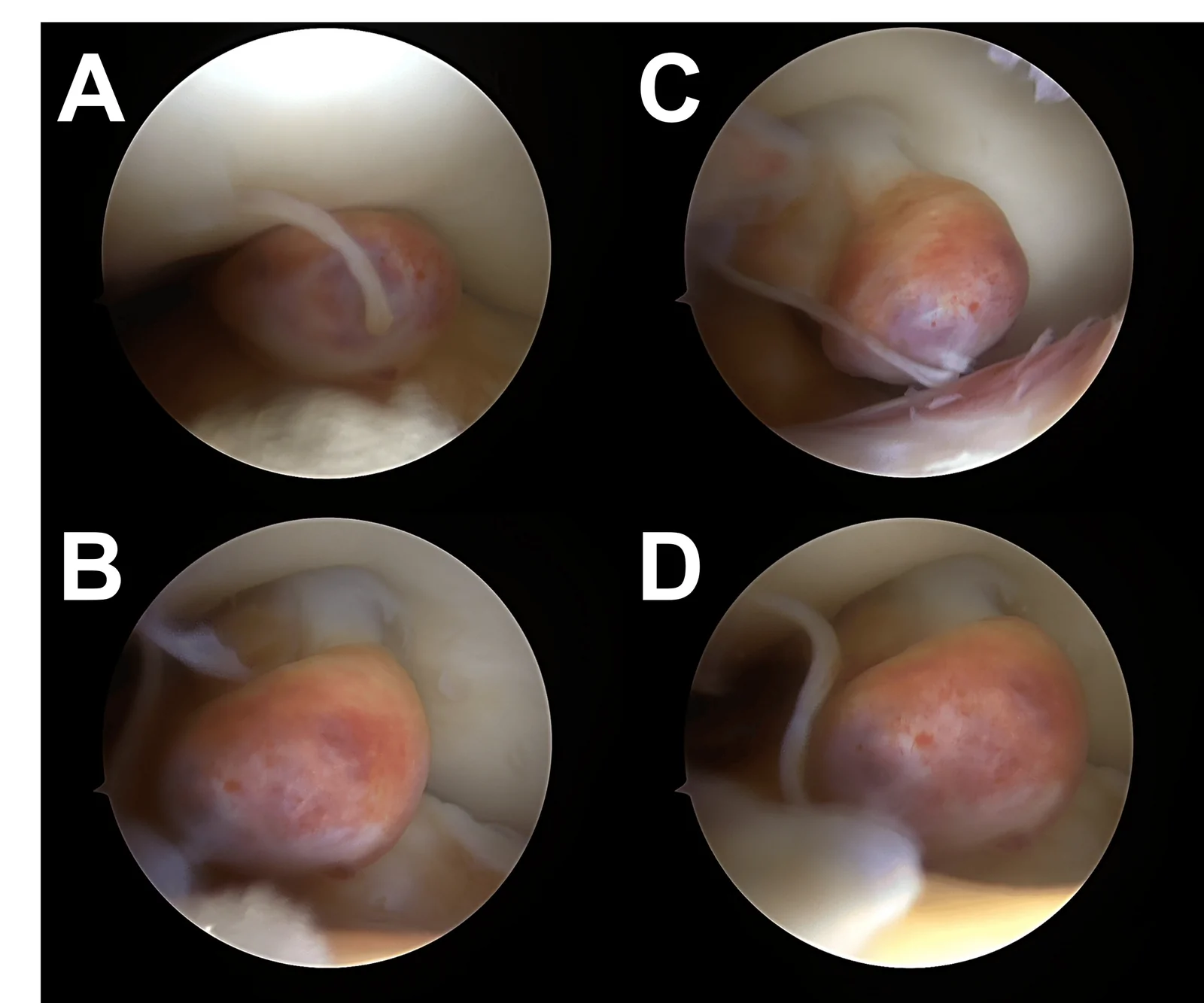
Arthroscopic visualization of the intra-articular loose body within the patellofemoral compartment. (A-D) Sequential arthroscopic views demonstrate a rounded fibrofatty loose body within the patellofemoral compartment. The lesion's gross morphology was considered suggestive of a presumed detached Hoffa's fat-pad remnant; however, no histopathologic confirmation was obtained, and direct continuity with the infrapatellar fat pad was not documented. A 30° arthroscope was used for visualization.

A prominent traumatic erythematous plica was also identified and resected using a motorized shaver and arthroscopic wand. Inflamed, hypertrophic-appearing synovial tissue was resected from the medial, lateral, and patellofemoral compartments with minimal bleeding. A lateral meniscal tear was treated with partial lateral meniscectomy using hand instruments and a 3.5-mm motorized shaver, with the remaining meniscus debrided to a smooth, stable border. The operative report did not further specify the tear morphology or subregion. It also noted an injured chondral area whose edges were probed for stability but did not provide a formal cartilage grade or precise location.

Local anesthetic consisting of equal parts 0.5% bupivacaine and 2% lidocaine was injected at the arthroscopic portal sites. The knee was irrigated, the portals were closed with 3-0 nylon sutures, and a sterile dressing was applied. The patient tolerated the procedure without intraoperative complication and was transferred to the recovery area in stable condition.

Postoperative management included weight-bearing as tolerated, anti-embolic stockings, and aspirin 325 mg daily for venous thromboembolism prophylaxis. Rehabilitation included straight-leg raises, quadriceps-setting exercises, prone hangs, and active-assisted knee flexion initiated in the recovery room.

At the six-week follow-up, the patient demonstrated marked short-term improvement following combined arthroscopic treatment. The Visual Analog Scale pain score decreased from 7/10 to 1/10, mechanical locking resolved, and range of motion improved from 10°-110° preoperatively to 0°-130°. No complication was reported at this visit. A validated knee-specific patient-reported outcome measure and longer-term follow-up were not available in the source materials used for this revision; therefore, no claims regarding recurrence or long-term efficacy are made.

## Discussion

This case describes an unusual fibrofatty intra-articular loose body identified in the patellofemoral compartment during arthroscopy for persistent anterior knee pain and mechanical symptoms. Loose bodies in the knee more commonly arise from osteochondral or meniscal tissue. Hoffa's disease, first described by Albert Hoffa in 1904, is characterized by impingement and inflammation of the infrapatellar fat pad and may progress to fibrosis, calcification, or ossification [1,6,7]. Published reports involving Hoffa's fat pad largely describe these processes or chondromatous and tumor-like lesions [3-5,8,9]. In contrast, a detached nonossified fibrofatty fragment presumed to arise from the fat pad is sparsely described, and a reliable incidence estimate for this specific entity is not available.

The differential diagnosis of an intra-articular loose body or mass in this location includes an osteochondral fragment, meniscal fragment, synovial chondromatosis, para-articular chondroma, lipoma arborescens or another lipomatous lesion, and tenosynovial giant-cell tumor. In this case, the lesion's gross arthroscopic appearance was fibrofatty rather than characteristically osseous or cartilaginous. Nevertheless, arthroscopic morphology alone cannot establish tissue origin, and histopathologic analysis was not obtained. In addition, the operative report did not document a discrete donor site or direct continuity with Hoffa's fat pad. Accordingly, the lesion is best described as a fibrofatty loose body presumed, rather than confirmed, to represent a detached Hoffa's fat-pad remnant.

MRI remains the primary imaging modality for characterizing Hoffa's fat-pad pathology and narrowing the differential diagnosis. In the present case, an MRI performed without intravenous contrast demonstrated a 1.4 × 0.5 × 0.2 cm flattened hypointense triangular lesion anterior to the ACL contiguous with adjacent Hoffa's fat-pad scarring on coronal PD, coronal fat-suppressed T2, sagittal PD, sagittal fat-suppressed T2, axial fat-suppressed PD, and sagittal T2 sequences. Despite these findings, MRI alone could not definitively establish whether the lesion represented detached fibrofatty tissue, scar tissue, displaced plica, or another intra-articular lesion. Arthroscopy therefore served as an important complementary diagnostic and therapeutic modality.

Arthroscopy allowed direct visualization and removal of the symptomatic intra-articular loose body while permitting treatment of concomitant pathology during the same procedure. However, several abnormalities were treated concurrently, including the loose body, a traumatic erythematous plica, inflamed hypertrophic-appearing synovium, and a lateral meniscal tear. The patient's postoperative improvement, therefore, cannot be attributed specifically to removal of the presumed Hoffa's fat-pad lesion. The appropriate interpretation is marked short-term improvement after combined arthroscopic treatment.

The six-week follow-up demonstrated improvement in pain, locking, and range of motion but does not establish long-term durability or absence of recurrence. In addition, no validated knee-specific outcome score was available. Other limitations include the absence of histopathologic confirmation, lack of documented direct continuity or donor site between the loose body and Hoffa's fat pad, incomplete characterization of the lateral meniscal tear and articular cartilage in the operative record, and currently incomplete MRI sequence characterization. These limitations reduce diagnostic certainty but do not diminish the educational value of recognizing an unusual fibrofatty intra-articular lesion and considering an appropriately broad differential diagnosis.

MRI and arthroscopy should therefore be viewed as complementary rather than interchangeable diagnostic tools. MRI remains important for lesion characterization and differential diagnosis, whereas arthroscopy may be appropriate when persistent mechanical symptoms warrant operative treatment and provides direct visualization with the opportunity for simultaneous management of intra-articular pathology [10,11].

## Conclusions

This case describes the arthroscopic identification and removal of an unusual intra-articular fibrofatty loose body in a 31-year-old man with persistent anterior knee pain and mechanical symptoms. Based on its gross arthroscopic appearance and location near the infrapatellar fat pad, the lesion was considered a presumed detached remnant of Hoffa's fat pad; however, its tissue of origin could not be definitively established because histopathologic examination was not obtained and direct continuity with the fat pad was not documented. The patient demonstrated marked short-term improvement in pain, mechanical symptoms, and range of motion following combined arthroscopic management of the loose body and associated intra-articular pathology. This case highlights the importance of maintaining a broad differential diagnosis for intra-articular loose bodies and of correlating MRI findings with operative observations while avoiding overstatement of tissue origin when histologic confirmation is unavailable.
